# An Established Th2-Oriented Response to an Alum-Adjuvanted SARS-CoV-2 Subunit Vaccine Is Not Reversible by Sequential Immunization with Nucleic Acid-Adjuvanted Th1-Oriented Subunit Vaccines

**DOI:** 10.3390/vaccines9111261

**Published:** 2021-11-01

**Authors:** Han Cao, Shengran Yang, Yunfei Wang, Ning Luan, Xingxiao Yin, Kangyang Lin, Cunbao Liu

**Affiliations:** Institute of Medical Biology, Chinese Academy of Medical Sciences and Peking Union Medical College, Kunming 650118, China; caohan@imbcams.com.cn (H.C.); YSR@imbcams.com.cn (S.Y.); wangyf@imbcams.com.cn (Y.W.); luanning@imbcams.com.cn (N.L.); xingxiao_y@imbcams.com.cn (X.Y.); linky6679@163.com (K.L.)

**Keywords:** SARS-CoV-2 subunit vaccine, Th1/Th2 orientation, alum adjuvant, nucleic acid adjuvant, prime-boost, homogenous boost immunization, heterologous boost immunization

## Abstract

A recently reported parallel preclinical study between a severe acute respiratory syndrome coronavirus 2 (SARS-CoV-2) mRNA vaccine and an inactivated SARS-CoV-2 vaccine adjuvanted with alum showed pulmonary immunopathology typical of eosinophil accumulation in a mouse pneumonia model for the latter, which implied a potential role of cellular immunity in the difference in the protection rate between these two forms of vaccines. For those who have been vaccinated with alum-adjuvanted subunit or inactivated SARS-CoV-2 vaccines, whether the Th2 responses that have been established and the absence of induced cellular immunity could be changed is an open question. Using two heterologous boosts with Th1-oriented CpG ODN-adjuvanted S1-based SARS-CoV-2 subunit vaccines for mice that were primed with two doses of Th2-oriented alum-adjuvanted S1-based SARS-CoV-2 subunit vaccines, we demonstrated that established Th2 orientation could not be reversed to Th1 orientation and that no cellular immunity was induced, which should have been induced if the boosting vaccines were used as the prime vaccines. These results remind us that if widely administered alum-adjuvanted SARS-CoV-2 vaccines cannot overcome the challenge of coronavirus disease 2019 (COVID-19) and that if cellular immunity is important for the efficacy of SARS-CoV-2 vaccines in the future, the choice of more powerful heterologous boosting vaccine forms that can induce cellular immunity should be considered very carefully before application.

## 1. Introduction

Since the first case of coronavirus disease 2019 (COVID-19) was reported in December 2019, severe acute respiratory syndrome coronavirus 2 (SARS-CoV-2) has infected 238 million people and caused 4.85 million deaths globally, as of the middle of October 2021 [1,2,3]. None of the medicines tried, including remdesivir, hydroxychloroquine and dexamethasone, have shown prominent effects in treating COVID-19 [4,5,6,7,8,9]. Considering its high infectivity, morbidity and mortality, vaccines are still the optimal choice to prevent SARS-CoV-2, except for public health measures.

Several forms of vaccines, including mRNA (i.e., BNT 162b2 developed by BioNTech, Mainz, Germany, and mRNA-1273 developed by Moderna, Cambridge, MA, USA) and inactivated vaccines (e.g., CoronaVac developed by Sinovac, Beijing, China), have been promptly and widely administered. While both mRNA vaccines showed protection rates above 90%, inactivated SARS-CoV-2 vaccines adjuvanted with alum showed protection rates between 50.7% and 83.5% in reported clinical phase III trials [10,11,12,13,14,15]. The difference in the protection rate could not be explained solely by the strength of the neutralization antibody responses induced because both the effective vaccines (including the mRNA vaccines mentioned above and the spike protein and adjuvant Matrix-M-based subunit vaccine NVX-CoV2373 developed by NOVAVAX, Gaithersburg, MD, USA) and inefficient alum-adjuvanted inactivated SARS-CoV-2 vaccines could induce neutralization antibody levels comparable to those in SARS-CoV-2-infected human convalescent serum (HCS) [16,17,18,19,20].

The induced cellular immunity may partially explain the difference in vaccine efficacy. While both subunit and inactivated SARS-CoV-2 vaccines adjuvanted with alum induced Th2-oriented responses with weak cellular immunity [20,21], NVX-CoV2373 depends on its Th1-oriented adjuvant Matrix-M to induce cellular immunity, and the Th1-biased responses of mRNA vaccines are highly dependent on the intracellular production of spike protein antigens and the innate immunity mobilization activity of mRNA itself [22,23,24,25]. In fact, during the development of vaccines for severe acute respiratory syndrome coronavirus (SARS-CoV-1) and Middle East respiratory syndrome coronavirus (MERS-CoV), preclinical studies showed that both subunit vaccines based on the spike proteins and inactivated vaccines that were adjuvanted with alum showed only partial protective effects, which were shown to reduce viral loads and pulmonary immune pathology characterized by eosinophil infiltration upon virus challenge [26,27,28,29]. Interestingly, adjuvants such as delta inulin and Toll-like agonists that induce prominent cellular immune responses could prevent or reduce excess eosinophilic infiltration in the lungs, ameliorate pulmonary immunopathology and enhance vaccine efficacy in mouse models [30,31].

Due to the consideration of vaccine efficacy elevation or safety concerns, additional vaccination schedules with homogenous or heterologous boost strategies for alum-adjuvanted inactivated SARS-CoV-2 vaccines are under consideration [32,33,34,35,36]. For those who have received full-schedule immunization with alum-adjuvanted subunit or inactivated SARS-CoV-2 vaccines, homogenous boosting is mainly aimed at elevating neutralization titers. As mentioned above, the induction of cellular immunity should be one of the key indicators for successful heterologous boosting (e.g., with mRNA vaccines or adenovirus vectored vaccines). In this study, we tested whether heterologous boosting of CpG oligodeoxynucleotide (ODN, the pathogen-related pattern stimulator of Toll-like receptors that induces Th1 responses to coadministered vaccine antigens)-adjuvanted subunit SARS-CoV-2 vaccines could reverse the Th2 responses that have been established by full-schedule immunization with alum-adjuvanted subunit SARS-CoV-2 vaccines and induce cellular immunity that has the potential to elevate vaccine efficacy, which implies careful choice of heterologous boosting vaccine forms to induce prominent cellular immunity.

## 2. Materials and Methods

### 2.1. Immunization of Mice

Female specific pathogen-free BALB/c mice (6 weeks of age, 14–17 g) were supplied, randomly divided into 6 mice in each group (*n* = 6) and maintained by the Central Service of the Institute of Medical Biology, Chinese Academy of Medical Sciences (IMB, CAMS).

**Figure 1:**
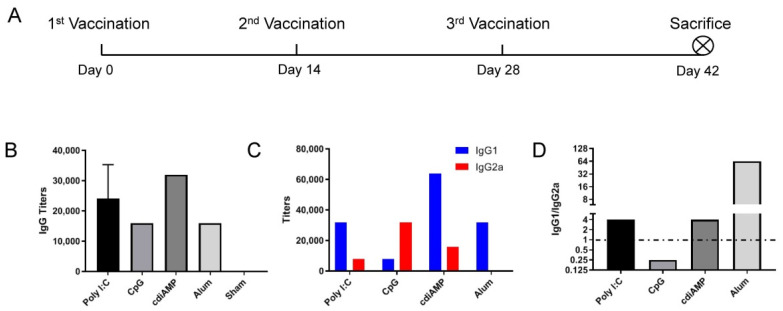
Humoral immune responses induced by different adjuvants with S1-based SARS-CoV-2 subunit vaccines. (**A**) Immunization schedule, *n* = 6; (**B**) S1-specific IgG titers of immune serum; (**C**) S1-specific IgG1 and IgG2a titers of immune serum; (**D**) IgG1/IgG2a ratio of immune serum. All of the above titers were detected in pooled sera from each immunized group, and the mean and standard deviation of duplicates are shown.

To determine the Th1/Th2 orientation induced by different adjuvants, HEK293 cell-expressed SARS-CoV-2 S1 proteins (Sino Biological Inc., Beijing, China) were diluted to 5 μg/mouse/dose in 25 μL of phosphate-buffered saline (PBS, pH 7.4) and mixed with the same volume of each of the following adjuvants: aluminum (Thermo Fisher, Eugene, OR, USA), 25 μg of phosphorothioate CpG ODN 2395 (InvivoGen, San Diego, CA, USA), 25 μg of low-molecular-weight polyinosinic–polycytidylic acid (poly I:C) (InvivoGen, San Diego, CA, USA), or 10 μg of cdiAMP (InvivoGen, San Diego, CA, USA). With 50 μL of PBS as a sham control, vaccines were administered intramuscularly in the thigh muscle three times at 2-week intervals (Figure 1A).

**Figure 2:**
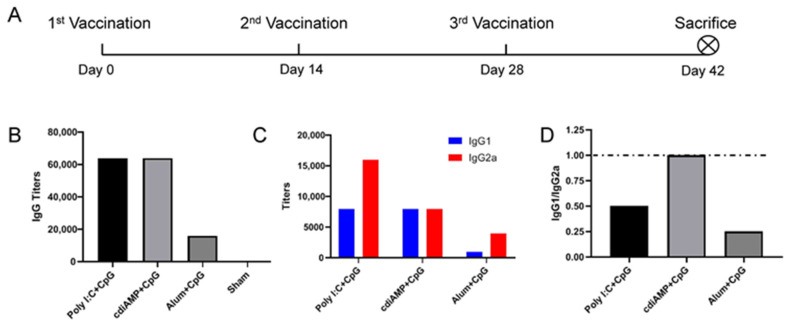
Humoral immune responses induced by different adjuvants mixed with CpG ODN in S1-based SARS-CoV-2 subunit vaccines. (**A**) Immunization schedule, *n* = 6; (**B**) S1-specific IgG titers of immune serum; (**C**) S1-specific IgG1 and IgG2a titers of immune serum; (**D**) IgG1/IgG2a ratio of immune serum. All of the above titers were detected in pooled sera from each immunized group, and the mean and standard deviation of duplicates are shown.

To determine the potential of Th1-oriented adjuvants to affect the activity of Th2-oriented adjuvants, the above adjuvants, including alum, poly I:C and cdiAMP, were added to 25 μg of CpG ODN 2395 and immunized with the same schedule as mentioned above (Figure 2A).

**Figure 3:**
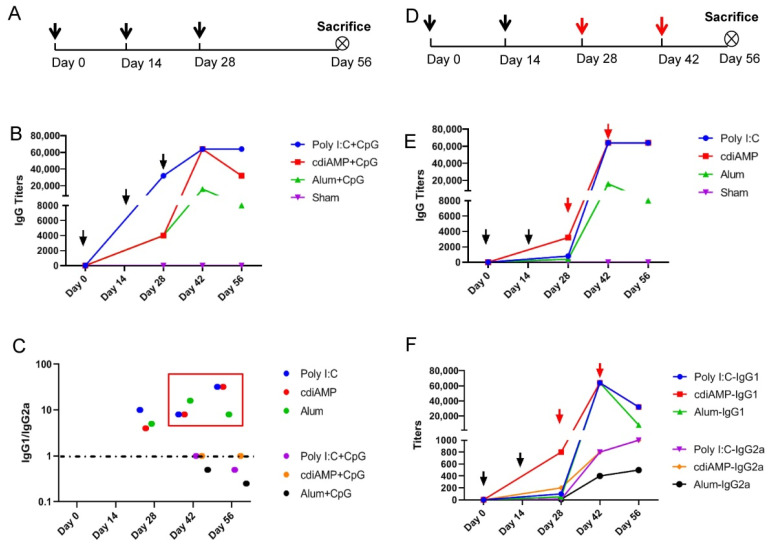
Comparison between the humoral immune responses induced by vaccines with Th2-oriented adjuvants mixed with CpG ODN and vaccines with Th2-oriented adjuvants then boosted by CpG ODN-adjuvanted vaccines. (**A**) Immunization schedule for vaccines with Th2-oriented adjuvants mixed with CpG ODN, *n* = 6; (**B**) S1-specific IgG titers of immune serum for immunization schedule (**A**); (**C**) IgG1/IgG2a ratio of immune serum for immunization schedule (**A**) including data shown as Poly I:C + CpG, cdiAMP + CpG, Alum + CpG and (**D**) including data shown as Poly I:C, cdiAMP, Alum; (**D**) Immunization schedule for vaccines with Th2-oriented adjuvants then boosted (boosting shown by red arrows) by CpG ODN-adjuvanted vaccines, *n* = 6; (**E**) S1-specific IgG titers of immune serum for immunization schedule (**D**); (**F**) S1-specific IgG1 and IgG2a titers of immune serum for immunization schedule (**D**). All of the above titers were detected in pooled sera from each immunized group, and the mean and standard deviation of duplicates are shown.

To determine whether Th1-oriented vaccines have the potential to reverse established Th2-oriented immunity, animals were primed twice at 2-week intervals with S1 immunogens adjuvanted with either alum, poly I:C or cdiAMP and boosted with 25 μg of CpG ODN 2395-adjuvanted S1 immunogens twice at 2-week intervals (Figure 3D). S1 immunogens adjuvanted with alum, poly I:C or cdiAMP that was supplemented with CpG ODN were administered three times at 2-week intervals, and these mice were used as controls (Figure 3A).

### 2.2. Enzyme-Linked Immunosorbent Assay (ELISA) of S1-Specific Antibody Titers

Before each immunization or on the sacrifice day after the final immunization, the mice were anesthetized with an intraperitoneal injection of tribromoethanol, and blood was collected via either tail veins or cardiac puncture. After clotting at 4 °C overnight, serum was collected by centrifugation at 3000 rpm for 10 min and pooled by group for further analysis. S1-specific IgG/IgG1/IgG2a titers were detected by ELISA, and the IgG1-to-IgG2a titer ratio was calculated to evaluate the Th1/Th2 balance as previously described [37].

### 2.3. Preparation of Splenocytes

Spleens were dispersed with a 70-μm cell strainer (BD, Indianapolis, IN, USA), and cells were collected by centrifugation at 1800 rpm for 5 min. After incubation with ammonium-chloride-potassium (ACK) lysis buffer at room temperature for 5 min, PBS was added to terminate red blood cell lysis. After centrifugation at 1800 rpm for 5 min at 4 °C, the splenocytes were suspended in PBS for enumeration, centrifuged again and resuspended in Roswell Park Memorial Institute (RPMI) 1640 medium with 10% *v*/*v* fetal bovine serum (both from Biological Industries, Kibbutz Beit-Haemek, Israel) and penicillin-streptomycin (Thermo Fisher) at a final concentration of 1 × 10^7^ cells/mL. Then, 100 μL of cells was added to each well of a 96-well plate (Corning, NY, USA) for further analysis.

### 2.4. Flow Cytometry

All following reagents were purchased from Biolegend, San Diego, CA, USA. A total of 1 × 10^6^ splenocytes were incubated with 10 μg/mL S1 proteins at 37 °C with 5% CO_2_ for 2 h, brefeldin A was added, and the splenocytes were incubated overnight under the same conditions to block cytokine release. After washing with staining buffer, 5 μg/mL anti-CD16/CD32 antibodies were added and the splenocytes were incubated at 4 °C for 10 min to block nonspecific binding of Fc receptors. After incubation with PC5.5-tagged anti-mouse CD4 and FITC-tagged anti-mouse CD8 antibodies at 4 °C for 30 min, the cells were fixed with fixation buffer in the dark at room temperature for 20 min. After washing with permeabilization wash buffer, PE-tagged anti-mouse IFN-γ antibodies were added, and the splenocytes were kept in the dark at room temperature for 30 min. After one wash with permeabilization wash buffer and one wash with PBS, the cells were gated (forward and site scatter, FSC/SSC), and samples with more than 20 000 events of CD4^+^ or CD8^+^ T cells were analyzed with a CytoFLEX flow cytometer (Beckman, Indianapolis, IN, USA) and FlowJo_V10 software (BD, Franklin Lakes, NJ, USA) [38].

### 2.5. Statistical Analysis

The antibody levels are shown as the mean and standard deviation. Data from the flow cytometry were analyzed using an unpaired *t* test. GraphPad Prism 8.0 (San Diego, CA, USA) was used for statistical analyses.

## 3. Results

### 3.1. CpG ODN Induces Th1 Skewing, and Alum Induces Th2 Skewing Responses to S1 Subunit SARS-CoV-2 Vaccines

After three immunizations (Figure 1A), all of the adjuvanted S1 vaccines induced the production of the corresponding S1-specific IgG antibodies (Figure 1B, titers of 24,000 for poly I:C, 16,000 for CpG ODN and alum and 32,000 for cdiAMP). Although CpG ODN and alum induced the production of comparable levels of IgG antibodies, the compositions of these antibodies were completely different (Figure 1C): while IgG2a titers were 4 times (32,000 vs. 8000) higher than IgG1 titers with CpG ODN-adjuvanted vaccines (a typical Th1-oriented response), IgG2a titers were almost undetectable compared with the titers of IgG1 subtype antibodies (500 vs. 32,000) in alum-adjuvanted vaccines (a typical Th2-oriented response). In fact, CpG ODN is the only adjuvant that induced IgG1/IgG2a titers lower than 1 (i.e., 0.25). While the IgG1/IgG2a ratio was as high as 64 for alum-adjuvanted vaccines, more balanced but still Th2-oriented responses were induced with poly I:C and cdiAMP adjuvants (IgG1/IgG2a = 4, Figure 1D).

### 3.2. Combination with CpG ODN Could Reverse Alum-Induced Th2 Responses to a Th1 Orientation

When CpG ODN was added to Th2-oriented adjuvants, it only doubled the antibody titers induced by poly I:C and cdiAMP and did not elevate alum-induced antibody titers after the same immunization schedule (Figure 2A,B). Notably, although CpG showed limited effects in inducing higher S1-specific total IgG antibody levels, it did elevate the proportion of IgG2a subtype S1-specific antibodies compared to those induced by Th2-oriented vaccines (Figure 2C). While the cdiAMP-induced Th2 response seemed to be slightly difficult to change (the IgG1/IgG2a ratio was equal to 1 instead of lower than 1, Figure 2D), which may be attributed to the highest IgG titers being induced by cdiAMP, CpG ODN reversed the Th2 response to the other two adjuvants to a typical Th1 orientation (the IgG1/IgG2a ratio changed from 4 to 0.5 for poly I:C and from 64 to 0.25 for alum).

### 3.3. Established Th2-Oriented Response to Alum-Adjuvanted SARS-CoV-2 Subunit Vaccines Is Irreversible by Sequential Immunization with CpG ODN-Adjuvanted Th1-Oriented Subunit Vaccines

A fourth immunization was ineffective in elevating antibody titers in the short term after the third immunization because IgG antibody levels induced by each adjuvanted vaccine reached a plateau stage after the third immunization. Two weeks after the third immunization on day 42, poly I:C- and cdiAMP-adjuvanted vaccines produced IgG antibody titers of 64,000 with (Figure 3B) or without (Figure 3E) the CpG ODN combination, and alum-adjuvanted vaccines produced IgG antibody titers of 16,000 with (Figure 3B) or without (Figure 3E) the CpG ODN combination. The presence of the CpG ODN combination showed limited ability to elevate the final IgG antibody titers after the third immunization, but seemed to accelerate the speed of antibody level induction, which was shown by the fact that IgG antibody titers were generally higher with each adjuvanted vaccine containing CpG ODN (Figure 3B) than with the corresponding adjuvanted vaccines without CpG ODN (Figure 3E) 2 weeks after the second immunization on day 28. In addition, antibody titers (IgG titers for alum- and cdiAMP-adjuvanted vaccines shown in Figure 3B, IgG titers for alum-adjuvanted vaccines shown in Figure 3E and IgG1 titers for all poly I:C-, cdiAMP- and alum-adjuvanted vaccines shown in Figure 3F) began to decline 2 weeks after the last (i.e., third or fourth) immunization.

After two doses (day 0 and day 14, shown by black arrows in Figure 3D) of a Th2-skewing vaccine (vaccines using poly I:C, cdiAMP or alum as adjuvants), all of the immune serum from immunized animals revealed the establishment of a Th2-oriented response (serum collected before the third immunization on day 28 that showed IgG1/IgG2a ratios higher than 1 in Figure 3C, including blue dots for poly I:C, red dots for cdiAMP and green dots for alum) that could not be reversed by one-dose or two-dose sequential immunization of CpG ODN adjuvanted Th1-oriented subunit vaccines (day 28 and day 42, shown by red arrows in Figure 3D), which was reflected by the fact that serum collected before the fourth immunization on day 42 and 2 weeks after the fourth immunization on day 56 both showed IgG1/IgG2a ratios higher than 1 (Figure 3C, boxed with red rectangles). Correspondingly, the immune sera from vaccines using these adjuvants supplemented with CpG ODN (from immunization schedules 3A) all showed IgG1/IgG2a equal to or lower than 1 (Figure 3C, including purple dots for poly I:C + CpG, orange dots for cdiAMP + CpG and black dots for alum + CpG).

In addition, Figure 3F shows that after one-dose sequential immunization of CpG ODN-adjuvanted Th1-oriented subunit vaccines, IgG2a and IgG1 levels increased disproportionately with each Th2-skewing vaccine, with IgG1 levels increasing faster. Interestingly, this tendency was reversed after the second-dose sequential immunization with CpG ODN-adjuvanted Th1-oriented subunit vaccines, with IgG2a levels increasing and IgG1 levels decreasing with each Th2-skewing vaccine.

### 3.4. Boosting of CpG ODN-Adjuvanted Th1-Oriented Subunit Vaccines Could Not Induce Cellular Immunity in Mice Primed with Alum-Adjuvanted SARS-CoV-2 Subunit Vaccines

**Figure 4:**
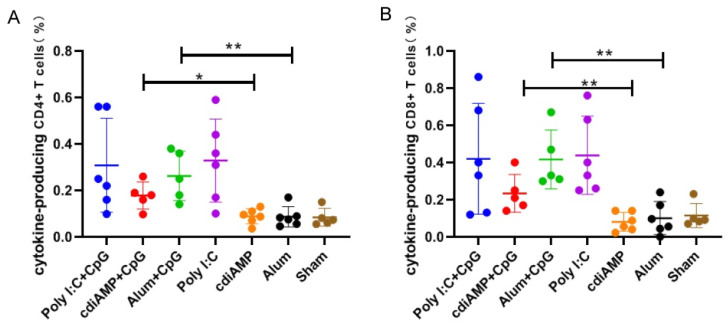
Flow cytometry assay for S1-specific IFN-γ-producing T cells. (**A**) Proportion of IFN-γ-producing CD4^+^ T cells among splenocytes after stimulation with S1; (**B**) Proportion of IFN-γ-producing CD8^+^ T cells among splenocytes after stimulation with S1. Data were analyzed using an unpaired *t* test with GraphPad Prism 8.0 (San Diego, CA, USA). *, *p* < 0.05; **, *p* < 0.01.

After the immunization schedule in Figure 3A, the Th2-oriented adjuvanted vaccines that were combined with Th1-skewing CpG ODN (including Poly I:C + CpG, cdiAMP + CpG and Alum + CpG) all showed significant cellular immunity, which was shown by a higher proportion of IFN-γ-producing CD4^+^ T cells (Figure 4A) and IFN-γ-producing CD8^+^ T cells (Figure 4B) compared with those of the sham controls. As a Th2-skewing adjuvant, poly I:C alone could induce not only potent humoral immunity, but also potent cellular immunity, which was confirmed in our previous studies [39]. For other Th2-skewing adjuvants that do not induce cellular immunity themselves, boosting immunization with Th1-skewing CpG ODN (shown as the immunization schedule in Figure 3D) could not induce cellular immunity, which was shown by the low proportion of IFN-γ-producing CD4^+^ T cells (Figure 4A) and IFN-γ-producing CD8^+^ T cells (Figure 4B) in the cdiAMP and Alum groups compared to the sham controls.

## 4. Discussion

Due to its high infectivity, morbidity and fatality and the unavailability of effective medicines, SARS-CoV-2 vaccines have been promptly and widely administered globally to combat the COVID-19 pandemic. While both FDA-approved mRNA vaccines showed protection rates above 90% in clinical phase III trials, inactivated SARS-CoV-2 vaccines adjuvanted with alum showed protection rates between 50.7% and 83.5% in corresponding trials [10,11,12,13,14,15]. Although the serological responses to SARS-CoV-2 vaccines may decline to suboptimal levels, as the serological responses to natural SARS-CoV-2 infection wanes within months, both the mRNA vaccines and inactivated vaccines were reported to induce the production of neutralization antibody levels comparable to those in the HCS, and both of the clinical trials were finished within months without observation of prominent antibody waning [16,17,18,19,20]. The difference in the protection rate could not be explained solely by the strength of the neutralization antibody responses induced, which made the homogenous boost strategies with inactivated vaccines to enhance the protection rate controversial, and heterologous boost strategies for inactivated vaccines are thus under consideration [34,35,36].

Cellular immunity should be considered one of the key indicators for effective boost strategies. In fact, one of the key differences between the less effective alum-adjuvanted SARS-CoV-2 vaccines and the successful vaccines (including lipid nanoparticle-encapsulated mRNA vaccines and a Matrix-M-adjuvanted subunit vaccine) is whether cellular immunity can be induced, which has been shown to be a Th1/Th2 response orientation in many studies [16,18,20,22,24,40,41,42,43]. Strictly speaking, Th1/Th2 response orientation evaluation by the induced antigen-specific IgG1-to-IgG2a antibody titer ratio cannot always indicate whether cellular immunity can be induced. The Th1-oriented response is definitely associated with the induction of cellular immunity, but cellular immunity is not necessarily missing in Th2-oriented responses. Previous studies have shown that while both subunit and inactivated SARS-CoV-1 vaccines adjuvanted with alum induced Th2-oriented responses with weak cellular immunity, corresponding antigens adjuvanted with pathogen-related pattern molecules, such as poly I:C and delta inulin, induced both Th2-oriented responses and strong cellular immunity [30,31]. More importantly, while alum-adjuvanted SARS-CoV-1 vaccines induced enhanced respiratory disease, as shown by eosinophilic infiltration in the lungs of mouse models upon virus challenge, corresponding poly I:C- and delta inulin-adjuvanted vaccines ameliorated pulmonary immunopathology and enhanced vaccine efficacy in these mouse models.

Among all of the adjuvants tested in this study, only CpG ODN induced Th1-oriented immune responses after vaccination, which implied successful cellular immunity production (Figure 1). While alum, cdiAMP, and poly I:C all induced Th2 responses, only poly I:C induced cellular immunity [31,39]. Interestingly, combination with CpG ODN reversed the alum- and cdiAMP-induced Th2 responses to the Th1 orientation, which was verified by both an antigen-specific IgG1-to-IgG2a antibody titer ratio lower than 1 (Figure 2) and production of antigen-specific IFN-γ-producing CD4^+^/CD8^+^ T cells (Figure 4, including Poly I:C + CpG, cdiAMP + CpG and Alum + CpG). Notably, once alum- and cdiAMP-induced Th2 responses were established after two doses of immunization, heterologous vaccination with CpG ODN-adjuvanted vaccines could not reverse this tendency, as shown by an antigen-specific IgG1-to-IgG2a antibody titer ratio higher than 1 (Figure 3C) and the absence of antigen-specific IFN-γ-producing CD4^+^/CD8^+^ T cells (Figure 4, including cdiAMP and Alum).

While pulmonary immunopathology accompanied by eosinophil accumulation in the respiratory tract upon SARS-CoV-2 infection has not been reported clinically with the widespread administration of either of the two COVID-19 mRNA vaccines and was not observed in preclinical studies of alum-adjuvanted inactivated SARS-CoV-2 vaccines in either murine or nonhuman primate pneumonia models, a recently reported parallel preclinical study between a SARS-CoV-2 mRNA vaccine and an inactivated vaccine adjuvanted with alum did show pulmonary immunopathology typical of eosinophil accumulation in mouse pneumonia models for the latter [40,41,42,44,45]. Considering the reportedly comparable strength of the neutralization antibody response induced between SARS-CoV-2 mRNA vaccines and alum-adjuvanted inactivated vaccines, the absence of cellular immunity accompanied by eosinophil-associated immunopathology may partially explain the lower protection of alum-adjuvanted inactivated SARS-CoV-2 vaccines in the real world, which has been certified as partial protection in preclinical studies of corresponding SARS-CoV-1 and MERS-CoV vaccines [20,26,27,28,29].

Although the vaccine-associated enhanced respiratory disease (VAERD) phenomenon accompanied by pulmonary eosinophil accumulation was similar, immunopathology associated with alum-adjuvanted inactivated coronavirus vaccines was not as lethal as that observed with alum-adjuvanted inactivated respiratory syncytial virus (RSV) vaccines [31,46]. This difference showed that while the protection rate of alum-adjuvanted inactivated SARS-CoV-2 vaccines against COVID-19 is only above 50%, with some of the mild cases possibly caused by mild immunopathology, the protection rate against clinically severe disease was nearly 90%, which demonstrated the key role of neutralizing antibodies in virus control or elimination upon primary contact, which is important compared with the potential immunopathology risk associated with severe COVID-19. For more tangible reasons, Th1-oriented adjuvants were taken to replace Th2 alum adjuvants in the development of inactivated SARS-CoV-2 vaccines, and BBV152 with pathogen-related pattern molecule adjuvants showed a protection rate of 77.8% against symptomatic disease under the background of disseminated delta variant strains [47]. If widely administered alum-adjuvanted SARS-CoV-2 vaccines cannot overcome the challenge of COVID-19 and cellular immunity is certified to be important for the efficacy of SARS-CoV-2 vaccines in the future, the choice of heterologous boosting vaccine forms should be considered carefully to induce prominent cellular immunity. Consistent with our results, a recent preclinical report showed that after two doses of an alum-adjuvanted inactivated SARS-CoV-2 vaccine, one dose of an adenovirus-vectored vaccine instead of one dose of an mRNA vaccine could induce cellular immunity that is higher than that induced with 2–3 doses of alum-adjuvanted inactivated SARS-CoV-2 vaccines.

## 5. Conclusions

Using two heterologous boosts of Th1-oriented CpG ODN-adjuvanted S1-based SARS-CoV-2 subunit vaccines for mice that were primed with two doses of Th2-oriented alum-adjuvanted S1-based SARS-CoV-2 subunit vaccines, we demonstrated that established Th2 orientation could not be reversed to Th1 orientation and that no cellular immunity was induced, which should have been induced if the boosting vaccines were used as the prime vaccines. If widely administered alum-adjuvanted SARS-CoV-2 vaccines cannot overcome the challenge of COVID-19 and cellular immunity is certified to be important for the efficacy of SARS-CoV-2 vaccines in the future, the choice of more powerful heterologous boosting vaccine forms that can induce cellular immunity should be considered very carefully before application.

## Data Availability

All data used during the study are available from the corresponding author by request.
